# Peridomestic Mammal Susceptibility to Severe Acute Respiratory Syndrome Coronavirus 2 Infection

**DOI:** 10.3201/eid2708.210180

**Published:** 2021-08

**Authors:** Angela M. Bosco-Lauth, J. Jeffrey Root, Stephanie M. Porter, Audrey E. Walker, Lauren Guilbert, Daphne Hawvermale, Aimee Pepper, Rachel M. Maison, Airn E. Hartwig, Paul Gordy, Helle Bielefeldt-Ohmann, Richard A. Bowen

**Affiliations:** Colorado State University, Fort Collins, Colorado, USA (A.M. Bosco-Lauth, S.M. Porter, A.E. Walker, L. Guilbert, D. Hawvermale, A. Pepper, R.M. Maison, A.E. Hartwig, P. Gordy, R.A. Bowen);; US Department of Agriculture, Fort Collins (J.J. Root);; The University of Queensland, St. Lucia, Queensland, Australia (H. Bielefeldt-Ohmann)

**Keywords:** severe acute respiratory syndrome coronavirus 2, SARS-CoV-2, coronavirus, viruses, coronavirus disease, COVID-19, respiratory infections, infections, cottontail rabbit, deer mouse, experimental infection, woodrat, squirrel, house mouse, peridomestic, mesocarnivore, raccoon, rodent, striped skunk, wildlife, zoonoses, United States

## Abstract

Wild animals have been implicated as the origin of severe acute respiratory syndrome coronavirus 2 (SARS-CoV-2), but it is largely unknown how the virus affects most wildlife species and if wildlife could ultimately serve as a reservoir for maintaining the virus outside the human population. We show that several common peridomestic species, including deer mice, bushy-tailed woodrats, and striped skunks, are susceptible to infection and can shed the virus in respiratory secretions. In contrast, we demonstrate that cottontail rabbits, fox squirrels, Wyoming ground squirrels, black-tailed prairie dogs, house mice, and racoons are not susceptible to SARS-CoV-2 infection. Our results expand the knowledge base of susceptible species and provide evidence that human–wildlife interactions could result in continued transmission of SARS-CoV-2.

The rapid global expansion of severe acute respiratory syndrome coronavirus 2 (SARS-CoV-2), which causes coronavirus disease (COVID-19), has been unprecedented in modern history. Although the original human infection(s) were potentially linked to wild animals in a wet market (*1*), human-to-human transmission is currently the dominant mechanism of viral spread. Peridomestic animals, which are represented by wild and feral animals living near humans, represent key species to evaluate for SARS-CoV-2 epidemiology for multiple reasons. First, given their common associations with humans and anthropogenically modified habitats, they represent the wildlife species with the greatest chance of exposure to the virus from humans (i.e., reverse zoonosis) or pets, such as cats. Second, should select peridomestic wildlife prove to be susceptible to the virus and have the capacity to replicate it to high viral titers, those species would have the potential to maintain the virus among conspecifics. Third, should some species possess the maintenance host criteria mentioned, they would represent wildlife species that would have the greatest chance (e.g., shedding ability and proximity to humans) to spread the virus back to humans. Wild rodents, cottontail rabbits (*Sylvilagus* sp.), raccoons (*Procyon lotor*), and striped skunks (*Mephitis mephitis*) can exhibit peridomestic tendencies in urban and suburban environments. Members of all these species/taxonomic groups have been shown to shed influenza A viruses after experimental inoculations (*2**–**4*), suggesting they might harbor productive infections when exposed to other human-pathogenic respiratory viruses.

Based upon protein analyses of amino acid residues of angiotensin-converting enzyme 2 (ACE2), transmembrane protease serine type 2, and spike protein, species susceptibility analyses suggested that, among other taxonomic groups, both carnivores and wild rodents are potentially high-risk groups (*5**–**7*). However, predicting susceptibility of specific species is more challenging. Looking at protein sequence analysis of ACE2 binding with the spike protein of SARS-CoV-2, one study indicated that raccoons could be ruled out as potential hosts for SARS-CoV-2 (*6*), and a different study based upon sequence analysis suggested that the western spotted skunks (*Spilogale gracilis*) had a low prediction of SARS-CoV-2 S binding propensity (*7*). Similarly, the same study also suggested that American mink (*Neovison vison*) have a similar prediction as western spotted skunks (*7*). However, over the past several months, outbreaks of SAR-CoV-2 in commercial mink farms have been reported in Europe and more recently in the United States (*8*,*9*). Respiratory problems, rapid transmission, or unusually high mortality rates have been reported for this species in various regions (*8*,*10*), which suggests that those analyses have limitations.

Because rodents are the largest and most diverse order of mammals, it is not surprising that the susceptibility of rodents to SARS-CoV-2 varies by species. To date, only a handful of rodent species have been evaluated as potential reservoir hosts or animal models for SARS-CoV-2, and the results largely indicate that outbred species, including laboratory animals, are at most only moderately affected. Most nontransgenic laboratory mice (*Mus musculus*) are resistant to infection, but transgenic humanized mice and hamsters, including Syrian hamsters (*Mesocricetus auratus*) and dwarf hamsters (*Phodopus* sp.), are highly susceptible (*11*,*12*); 1 report described Roborovki dwarf hamsters becoming diseased and dying within 3 days of exposure (*13*). Other species, including deer mice (*Peromyscus maniculatus*), become infected and shed low titers of virus, but the infection is subclinical (A. Fagre, Colorado State University, pers. comm., 2020 Aug 7). Considering that there are >1,700 species of rodents worldwide, many of which exist closely at the human–wildlife interface, there remain many unanswered questions about SARS-CoV-2 and wild rodents.

Various lagomorphs exist as pets, livestock, and peridomestic wildlife, and as such are in a prime position to come into contact with SARS-CoV-2‒infected humans. In 1 study, New Zealand white rabbits were experimentally infected and shed infectious virus for <7 days without signs of clinical disease (*14*). Wild rabbits, particularly cottontails in the United States, are prolific and commonly found around human dwellings, farms, and commercial buildings. Furthermore, as with rodents, wild rabbits are likely to be predated upon by domestic and wild felids and canids. Thus, the susceptibility of these animals must be determined to interpret the risk posed to them and by them from infection with SARS-CoV-2.

Among carnivores, felids and mustelids have been frequently linked to SARS-CoV-2 infections since the early stages of the pandemic. Domestic cats are highly susceptible to SARS-CoV-2 and are capable of transmitting the virus to other cats, suggesting that they could also potentially transmit virus to other animals (*15**,**16*). Although striped skunks are currently considered to be mephitids, they are highly related to mammals within the family Mustelidae and were formerly classified as mustelids. Thus, on the basis of findings for SARS-CoV-2 susceptibility in various mustelids, we determined that the closely related mephitids are a logical candidate to evaluate for replication of this virus. Raccoons are notoriously associated with human environments and frequently interact with human trash and sewage; these interactions have which has been proposed as a potential indirect means for infected humans to transmit SARS-CoV-2 to mammalian wildlife (e.g., raccoons and select mustelids) (*17**–**19*). Thus, it is essential to determine the relative susceptibility of these common peridomestic carnivores and assess the likelihood that they could propagate infection.

In this study, we assessed 6 common peridomestic rodent species for susceptibility to SARS-CoV-2: deer mice, wild-caught house mice (*Mus musculus*), bushy-tailed woodrats (aka pack rats; *Neotoma cinerea*), fox squirrels (*Sciurus niger*), Wyoming ground squirrels (*Urocitellus elegans*), and black-tailed prairie dogs (*Cynomys ludovicianus*). These rodents are common in many parts of the United States, several of them frequently come into close contact with humans and human dwellings, and some are highly social animals, thus increasing the likelihood of pathogen transmission among conspecifics. In addition, we evaluated 3 other common peridomestic mammals: cottontail rabbits, raccoons, and striped skunks.

## Materials and Methods

### Animals

We evaluated the following mixed-sex animals for susceptibility to SARS-CoV-2: deer mice, house mice, bushy-tailed woodrats, Wyoming ground squirrels, black-tailed prairie dogs, fox squirrels, cottontail rabbits, striped skunks, and raccoons. Deer mice, house mice, and bushy-tailed woodrats were trapped by using Sherman traps (https://www.shermantraps.com) baited with grain. Wyoming ground squirrels, fox squirrels, black-tailed prairie dogs, and cottontails were trapped using Tomahawk live traps (https://www.livetrap.com) (e.g., 7 in × 7 in × 20 in or 7 in × 7 in × 24 in). All trapping was conducted in northern Colorado (Larimer, Jackson and Weld Counties) in accordance with Colorado wildlife regulations and with appropriate permits and Institutional Animal Care and Use Committee protocols in place. Skunks and raccoons were purchased from a private vendor. Animals were housed in an Animal Biosafety Level 3 facility at Colorado State University, in rooms (12 ft × 18 ft) that had natural lighting and controlled climate. Mice, black-tailed prairie dogs, and Wyoming ground squirrels were group housed by species with access to water and food ad libitum. All other animals were housed individually with access to food and water ad libitum.

Rodents were fed Teklad Rodent Diet (Enviro, https://www.envigo.com) supplemented with fresh fruit and occasional nuts. Rabbits were fed Manna Pro alfalfa pellets (https://www.mannapro.com) supplemented with grass hay and apples. Skunks and raccoons were fed Mazuri Omnivore Diet (https://www.mazuri.com) supplemented with fresh fruit and occasional eggs. Raccoons, striped skunks, and black-tailed prairie dogs were implanted with thermally sensitive microchips (Bio-Thermo Lifechips, http://destronfering.com) for identification and temperature measurement and deer mice were ear notched; all other animals were identified by cage number or distinct markings.

### Virus

We obtained SARS-CoV-2 strain WA1/2020WY96 from BEI Resources (https://www.beiresources.org), passaged it twice in Vero E6 cells, and prepared stocks frozen at −80°C in Dulbecco modified Eagle medium containing 5% fetal bovine serum and antimicrobial drugs. We titrated the virus stock on Vero cells by using a standard double overlay plaque assay (*15*) and counted plaques 72 hours later to determine PFUs/mL.

### Virus Challenge

Before challenge with SARS-CoV-2, we lightly anesthetized most animals as needed with 1–3 mg/kg xylazine and 10–30 mg/kg ketamine hydrochloride and collected a blood sample just before inoculation (day 0). We administered virus diluted in phosphate-buffered saline to all species into the nares by using a pipette (50 μL for deer and house mice, 100 μL for bushy-tailed woodrats, and 200 μL for all other species) and observed animals until they were fully recovered from anesthesia. Virus back-titration was performed on Vero cells immediately after inoculation, confirming that animals received 4.5–4.9 log_10_ PFU of SARS-CoV-2.

### Sampling

We used groups of 3 animals from each species (2 ground squirrels) for preliminary studies to evaluate viral shedding and acute pathologic changes. For these animals, we obtained oral swab specimens prechallenge and on days 1–3 postchallenge, at which time animals were euthanized and the following tissues harvested for virus isolation and formalin fixation: trachea, nasal turbinates, lung, heart, liver, spleen, kidney, small intestine, and olfactory bulb. The exception to this process was raccoons, for which we euthanized only 1 animal at day 3; we kept the remaining 2 raccoons through day 28 to evaluate serologic response. We swabbed the remaining 3–6 animals/selected species daily from days 0–5 and 7 to further evaluate duration of viral shedding (if any). We sedated striped skunks and raccoons for all sampling and collected a nasal swab specimen in addition to the oral swab specimen. We evaluated tissues harvested from animals euthanized on day 7 as for the day 3 animals. We euthanized the remaining animals at 28 days postinfection (dpi), harvested tissues for histopathologic analysis, and collected serum for serologic analysis. We provide the necropsy scheme for each species (Table).

**Table:** Wildlife species evaluated for experimental infections with SARS-CoV-2 and day animals were euthanized*

Animals	No. euthanized at 3 dpi	No. euthanized at 7 dpi	No. euthanized at 28 dpi
Deer mice, n = 9	3	3	3
House mice, n =6	3	0	3
Bushy-tailed woodrats, n = 6	3	0	3
Fox squirrels, n = 3	3	0	0
Wyoming ground squirrels, n = 2	2	0	0
Black-tailed prairie dogs, n = 9	3	3	3
Cottontails, n = 3	3	0	0
Raccoons, n = 3	1	0	2
Striped skunks, n = 6	3	0	3

*dpi, days postinfection; SARS-CoV-2, severe acute respiratory syndrome coronavirus 2.

### Clinical Observations

We clinically evaluated all animals daily and included assessment for temperament and any clinical signs of disease, such as ocular discharge, nasal discharge, ptyalism, coughing/sneezing, dyspnea, diarrhea, lethargy, anorexia, and moribund status. The stress of handling wild animals for sampling precluded the ability to obtain accurate body temperature measurements; as such, we excluded temperature in these preliminary studies for all species except skunks and raccoons, which were implanted with thermal microchips and could be measured under sedation during sampling.

### Viral Assays

We performed plaque reduction neutralization assays as described (*15*). Serum samples were heat-inactivated for 30 min at 56°C, and 2-fold dilutions were prepared in Tris-buffered minimal essential medium containing 1% bovine serum albumin starting at a 1:5 dilution and aliquoted onto 96-well plates. An equal volume of virus was added to the serum dilutions and incubated for 1 hour at 37°C. After incubation, serum‒virus mixtures were plated onto Vero monolayers as described for virus isolation assays. We screened serum samples for antibodies specific to SARS-CoV-2 to ensure seronegative status before inoculation by using a cutoff value <50% viral neutralization. We recorded antibody titers as the reciprocal of the highest dilution in which >90% of virus was neutralized.

### Serologic Analysis

We performed plaque reduction neutralization assays as described (*15*). We heat-inactivated serum samples for 30 min at 56°C, and prepared 2-fold dilutions in Tris-buffered minimal essential medium containing 1% bovine serum albumin starting at a 1:5 dilution and aliquoted onto 96-well plates. We added an equal volume of virus to the serum dilutions and incubated the serum dilutions for 1 hour at 37°C. After incubation, we plated serum‒virus mixtures onto Vero monolayers as described for virus isolation assays. We screened serum samples for antibodies specific to SARS-CoV-2 to ensure seronegative status before inoculation by using a cutoff value <50% viral neutralization. We recorded antibody titers as the reciprocal of the highest dilution in which >90% of virus was neutralized.

### Quantitative Reverse Transcription PCR

We picked plaques from culture plates from each positive animal to confirm SARS-CoV-2 viral shedding. We extracted RNA by using QiaAmp Viral RNA Mini Kits (QIAGEN, https://www.qiagen.com) according to the manufacturer’s instructions. We performed reverse transcription PCR (RT-PCR) by using the E_Sarbeco primer probe sequence described by Corman et al. (*20*) and the Superscript III Platinum One-Step qRT-PCR System (Invitrogen, https://www.thermofisher.com) with the following modification: the initial reverse transcription was at 50°C. RNA standards for PCR were obtained from BEI Resources.

### Histopathologic Analysis

We fixed animal tissues in 10% neutral-buffered formalin for 12 days and transferred them to 70% ethanol before processing for paraffin embedding and sectioning for staining with hematoxylin and eosin. Slides were read by a veterinary pathologist blinded to the treatments.

## Results

### Viral Shedding

Of the 9 species evaluated, 3 (deer mice, bushy-tailed woodrats, and striped skunks) shed infectious virus after challenge (Figure). Deer mice, which have previously been demonstrated to shed infectious SARS-CoV-2 experimentally (A. Fagre, Colorado State University, pers. comm., 2020 Aug 7), shed virus orally for <4 days and virus was isolated from lungs (3/3) and trachea (2/3) of animals tested at 3 dpi. All 9 inoculated deer mice shed virus on at >2 of the first 4 days after infection and had peak titers of 3.1 log_10_ PFU/swab specimen. Bushy-tailed woodrats shed virus orally for <5 days postinoculation (6/6), and virus was isolated from turbinates (2/3), trachea (1/3), and lung (1/3) from animals that underwent necropsy on 3 dpi. Peak titers from bushy-tailed woodrats reached 3.0 log_10_ PFU/swab specimen 3 dpi. The single bushy-tailed woodrat for which infectious virus was isolated from the lungs only shed 1.3 log_10_ PFU/swab specimen orally on the day of necropsy, but the lungs contained 5.2 log_10_ PFU/g of virus.

**Figure:**
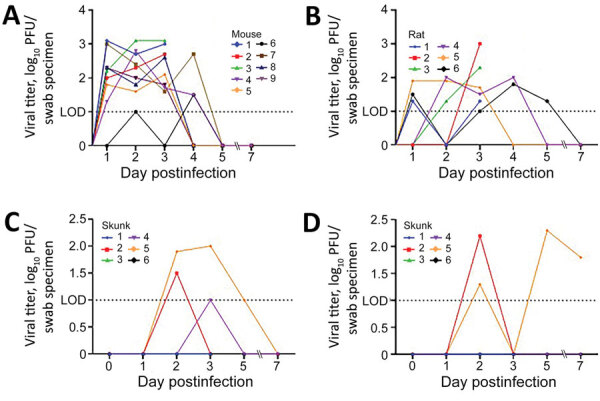
Oropharyngeal shedding of severe acute respiratory syndrome coronavirus 2 in deer mice (A), bushy-tailed woodrats (B), and striped skunks (C) and nasal shedding in striped skunks (D). LOD = 1 log_10_ PFU. LOD, limit of detection.

Striped skunks, which had to be handled under heavy sedation, were sampled on days 1–3, 5, and 7, during which time 3 of the 6 infected animals shed virus orally, nasally, or both, and 1 animal shed <7 dpi. Of the 3 skunks that underwent necropsy on 3 dpi, 2 had infectious virus in the turbinates but not in other tissues tested. One of those 2 animals had 3.2 log_10_ PFU/g of virus in the turbinates but did not shed detectable virus nasally or orally before euthanasia. In general, viral titers were slightly higher in nasal samples than oral samples, but overall peak titers in skunks were relatively low, with oral titers reaching 2 log_10_ PFU/swab specimen and nasal titers reaching 2.3 log_10_ PFU/swab specimen. All animals that had plaque assay‒positive samples were confirmed as having SARS-CoV-2 by RT-PCR. Similarly, all animals that were negative by plaque assay were confirmed as negative for viral shedding by RT-PCR.

### Seroconversion

All animals were seronegative against SARS-CoV-2 at the time of inoculation. On the basis of the lack of evidence of infection and the overall difficulty of maintaining wildlife, we opted not to hold subsets of squirrels or rabbits for additional time to assess seroconversion. We assessed neutralizing antibody titers in all animals euthanized at 28 dpi, which included deer mice, house mice, bushy-tailed woodrats, black-tailed prairie dogs, raccoons, and striped skunks. All species that had detectable viral infections (deer mice, skunks, and bushy-tailed woodrats) also had neutralizing antibodies develop whereas the other species (house mice, raccoons, and black-tailed prairie dogs) did not. Deer mice and bushy-tailed woodrats reached or exceeded titers of 1:80, the 2 skunks that shed infectious virus reached or exceeded titers of 1:160, and the single skunk that did not shed virus had a titer of 1:10 at 28 dpi. We did not test animals euthanized at 3 dpi for seroconversion because previous investigations have demonstrated that neutralizing antibodies are typically not detectable during acute infection (*21*).

### Clinical Disease

None of the animals exhibited clinical signs of disease (see Materials and Methods) at any time during the study. Skunks and raccoons, which were sedated for sampling procedures, did not display increased elevated temperatures at those times. In addition to monitoring clinical signs, we monitored behavior by observing animals through double-paned glass and assessing eating and response to provided enrichment (playing with toys, eating treats, using hides). None of the animals behaved abnormally after infection compared with the acclimation period.

### Pathology

None of the animals had gross lesions at the time of necropsy. At histopathologic examination of tissues harvested 3 dpi, rare, small foci of mild macrophage and neutrophil infiltration were noted in the lungs of 2 woodrats and 2 deer mice with one of the deer mice also having mild vasculitis. Two skunks had well-developed bronchioles associated lymphoid tissue, but inflammation was not apparent in the lungs or other tissues.

## Discussion

COVID-19 has had a major impact on the human population globally, but so far little is known about how SARS-CoV-2 virus affects wildlife. Domestic cats and dogs have repeatedly been shown to be infected by SARS-CoV-2, but with few exceptions these infections are subclinical or animals show development of mild clinical disease (*15*,*22*,*23*). Conversely, farmed mink are not only susceptible to infection but also can have fulminating fatal disease develop (*10*,*24*). In contrast, ferrets, which are closely related to mink, shed virus after infection, but the infection is subclinical (*25*). Raccoon dogs, which were heavily implicated in the severe acute respiratory syndrome outbreak during 2002–2004, are susceptible to SARS-CoV-2 infection, but infections remain subclinical (*26*). Experimentally, deer mice can be infected and shed the virus by oral secretions, as demonstrated by this study and others (A. Fagre, Colorado State University, pers. comm., 2020 Aug 7). However, other mice, including wild house mice and nontransgenic laboratory strains of this species, are not susceptible to infection by SARS-CoV-2 (*27*).

Studies in which bats and select small mammals were experimentally exposed to SARS-CoV-2 showed that some species (i.e., fruit bats [*Rousettus aegyptiacus*] and tree shrews [*Tupaia belangeri*]) are capable of minimal viral replication, but others (big brown bats [*Eptesicus fuscus*]) do not become infected, which suggests that although the virus might have originated in bats, they are unlikely to serve as reservoir hosts (*28**–**30*). The confounding clinical response to infection between closely related species makes predicting impacts on wildlife and their potential for reservoir maintenance difficult. Despite best attempts to predict host susceptibility on the basis of receptor similarity or other modeling approaches, experimental infections remain the standard for evaluating the susceptibility of an animal to infection and following the course of disease.

Our results demonstrate that several common peridomestic wildlife species, including deer mice, bushy-tailed woodrats, and striped skunks, are susceptible to SARS-CoV-2 infection and can shed infectious virus. Our results and the results of others indicate that so far, most exposed wildlife species show development of mild to no clinical disease and either did not shed virus or shed low levels for short durations (*26**,**28**–**30*). These experimental infections suggest that we can rule out several common rodents, selected wild lagomorphs, and raccoons as potential SARS-CoV-2 reservoirs. However, there are limitations to these experimental models, namely that the animals in our studies were directly exposed to high doses (e.g., 5 log_10_ PFU) of virus, which is unlikely to be representative of an exposure in nature. In addition, experimental infections using low numbers of apparently healthy, immunocompetent animals do not generate sufficient data to fully characterize the risk posed to animals of varying ages and health status. However, results of this study and results of others, combined with the dramatic response to infection seen in certain species, such as mink, indicate that SARS-CoV-2 might infect infecting wildlife, establishing a transmission cycle, and becoming endemic in nonhuman species. In particular, the relatively high titers observed in select woodrat tissues (e.g., 5.2 log_10_ PFU/g of lung) suggests that a predator‒prey transmission scenario among this rodent species and various small wild and domestic carnivore species is plausible, and experiments designed to capture oral transmission between prey and predator are a logical next step in determining the likelihood of this scenario.

The major outcomes of such an event include direct threat to the health of wildlife and establishment of a reservoir host, which could complicate control measures of this virus in human populations. Experimental studies to identify and characterize responses of species to SARS-CoV-2 infection help scientists classify those species that are at highest risk and enable implementation of prevention measures. For example, because deer mice and bushy-tailed woodrats are commonly found in barns and sheds near humans, when cleaning out sheds or attempting to rodent-proof barns, persons should consider wearing appropriate personal protective equipment to prevent exposure to the pathogens that rodents carry, as well as to prevent exposing wildlife to SARS-CoV-2. Persons whose occupations put them in contact with susceptible animals (biologists, veterinarians, rehabilitators) and COVID-19 patients who own cats and dogs should practice extra precaution when interacting with animals, including minimizing their pet’s exposure to wildlife. Of note, a photo-monitoring study provided evidence that striped skunks can commonly use the same urban cover types (e.g., outbuildings and decks) as domestic cats (*31*). Intentionally available pet food and spilled bird feed, which were 2 of the attractants evaluated, produced instances where skunks and domestic cats were documented to be on study sites simultaneously or nearly simultaneously, which could lead to interspecies transmission of SARS-CoV-2.

Wildlife and SARS-CoV-2 are intricately involved, from the initial spillover event to potential reverse zoonotic transmission, and we will undoubtedly continue to discover more susceptible species as the search for zoonotic reservoirs continues. COVID-19 is just the latest in a series of examples of how the human–wildlife interface continues to drive the emergence of infectious disease. Using experimental research, field studies, surveillance, genomics, and modeling as tools for predicting outbreaks and epidemics should help provide the knowledge base and resources necessary to prevent future pandemics.
